# Effect of digital multimedia on the adoption of agricultural green production technology among farmers in Liaoning Province, China

**DOI:** 10.1038/s41598-024-64049-w

**Published:** 2024-06-07

**Authors:** Xueying Yu, Guojun Sheng, Dongshi Sun, Rui He

**Affiliations:** 1 Dalian Neusoft University of Information, Dalian, China; 2 Wenzhou Business College, Wenzhou, China

**Keywords:** Digital multimedia, Green agriculture, Technology acceptance model, Environmental economics, Sustainability

## Abstract

Agricultural green production technology (AGPT) is essential for the sustainable development of agriculture, yet adoption rates among farmers are often low due to limited access to education and information. Based on the extended technology acceptance model, this study takes Liaoning, a major agricultural province in China, as a representative region to explore how digital multimedia influences the adoption of AGPT by farmers. The findings show that the perceived ease of use of these technologies significantly enhances farmers' intentions to adopt, while perceived risks detract from these intentions. Although digital multimedia effectively promotes AGPT by improving its perceived ease of use and usefulness, its role in mitigating perceived risks is minimal. Based on these results, we recommend that the government improve the quality of information on digital platforms by involving experts in the field and offer specific digital marketing training to potential users to increase the adoption of sustainable agricultural technologies.

## Introduction

Agriculture plays a central role in the national economy of China, one of the world's most populous countries. However, intensive management practices in traditional agricultural production often lead to environmental pollution^1^, which poses a severe threat to the sustainable development of agriculture. Consequently, promoting agricultural green production technology (AGPT) has become necessary to manage agricultural pollution^2^. AGPT refers to the adoption of environmentally friendly, efficient, and sustainable technologies and methods, including improved agricultural techniques and sustainable production practices. It promotes comprehensive, sustainable economic, societal, and environmental growth by reducing chemical inputs, optimizing resource allocation, and enhancing ecosystem services^3–5^. The implementation of AGPT varies across countries. Statistics on the economic benefits derived from crop rotations in the midwestern United States indicate that farmers diversify their sources of income and improve soil conditions by employing diversified crop rotations, which enhance resilience to drought and other challenging conditions^6^. Moreover, organic farming carried out in Australia effectively reduces the use of chemical fertilizers and pesticides^7^, adding value to agricultural products while protecting soil and water quality and reducing greenhouse gas emissions^8^. In addition, the straw return technology implemented in India and Brazil has been proven to effectively enhance soil fertility and crop yield and reduce air pollution from straw burning^9,10^. However, due to limited education, limited information channels, and entrenched traditional agricultural practices, many Chinese farmers still have a low level of awareness of AGPT^11^. This reluctance or lack of knowledge about techniques has hindered the expected impact of AGPT in practice^12^. Therefore, exploring the key factors influencing farmers' awareness of AGPT is not only central to promoting green agricultural development but also a prerequisite for achieving policy goals.

The evolution of digital multimedia has transformed the ways in which information is conveyed, making educational content more accessible and engaging^13^. This transformation has also occurred in the realm of agricultural technology education and practical application. For example, in Minnesota, agricultural educators have begun employing digital multimedia technologies to enhance the delivery of modern agricultural knowledge^14^. Similarly, in China, several successful cases demonstrate how digital multimedia technology effectively supports farms primarily operated by family members^15^, enabling them not only to maintain commercialization and scalability but also to achieve efficient, intensive operations. However, further scrutiny of the literature underscores that while digital multimedia’s role in fostering information dissemination is acknowledged, there is insufficient exploration of how specific types of digital content—such as targeted awareness campaigns and personalized advisories—affect farmers' attitudes and behaviours towards adopting AGPT. Crucially, there appears to be a considerable research void concerning the psychological and sociocultural barriers that impede the effectiveness of digital interventions in rural agricultural settings in China. Therefore, this paper aims to fill these gaps by proposing a new research model that investigates the impact of digital multimedia on farmers' willingness to adopt AGPT, examining the roles of various forms of digital content and their effectiveness in altering agricultural practices in culturally and economically diverse settings.

Consequently, this paper aims to extend the technology acceptance model (TAM) by incorporating perceived risk to examine the influence of digital multimedia on farmers' adoption of AGPT in China. Liaoning Province is selected as the study site due to its status as a major grain-producing region, which underscores its vital role in China’s agricultural sector. Additionally, the local government's active promotion of environmentally friendly agricultural practices provides an exemplary context for studying the impact of digital multimedia on the adoption of green technologies. The objectives of this study are as follows:

1. To propose a novel theoretical model that integrates perceived risk into the TAM, analysing the impact of digital multimedia on farmers' willingness to adopt AGPT.
2. To conduct a field survey in the main grain-producing areas of Liaoning Province using descriptive statistical analysis to gather data on farmers' characteristics and structured surveys designed to collect detailed information on their acceptance of AGPT.
3. To test the effectiveness of the extended TAM in predicting and explaining the adoption intentions of farmers towards AGPT using structural equation modelling.
4. To analyse survey data to elucidate how digital multimedia influences farmers' adoption of AGPT and to offer insights for enhancing future adoption rates.

Building on the stated objectives, the primary contributions of this study are as follows: First, this paper investigates how digital multimedia can bridge the informational divide and foster technology acceptance. To our knowledge, this study is among the first to systematically analyse and quantify the impact of digital multimedia as a significant impact factor in the adoption of AGPT within the agricultural context of Liaoning Province. Second, this research enriches the literature by successfully integrating new variables into the TAM and provides a more holistic understanding of the factors influencing farmers' decision-making processes regarding AGPT adoption, particularly in the context of the influence of digital multimedia. Third, the analysis of survey data offers new insights into the dynamics between digital multimedia and farmers' adoption intentions. The results reveal that digital multimedia significantly enhances the perceived ease of use and usefulness of AGPT while also illustrating that perceived risk can dampen adoption intentions. These findings provide a robust framework for future research and policy formulation aimed at increasing the adoption of sustainable agricultural technologies.

The rest of this paper is organized as follows: Section “Methodology and Data” constructs the model, adding the perceived risk variable to the TAM and proposing hypotheses based on preliminary theoretical foundations. Section "Results" presents the research results, the design of a survey questionnaire based on preliminary measurement items and the analysis of the collected sample data using structural equation modelling to verify the effectiveness of the proposed research model. Section "Discussion and Implications" discusses the results, interpreting the theoretical and practical significance of the study in light of the hypothesis testing results. Finally, Section "Conclusions" concludes the paper and presents potential future research directions.

## Literature review

### Advances in AGPT research

The concept of agricultural green production technology (AGPT) originated in the mid-twentieth century when scholars and policymakers began to recognise the impact of agricultural activities on ecosystems. During the 1960s and 1970s, the Green Revolution significantly increased food production through the introduction of high-yield crop varieties, chemical fertilisers, and irrigation techniques, addressing famine in many regions. However, the intense agricultural practices and heavy use of chemical fertilisers also led to adverse environmental effects, such as soil degradation and water pollution^16^. To address these issues, agricultural research shifted focus towards sustainable development and eco-friendly technologies, leading to the emergence of green production technologies that aimed to transform traditional agriculture into a system characterised by high productivity, efficient resource use, and minimal environmental impact^17^. Over the past 30 years, agricultural technological innovations have propelled the green development of agriculture globally, especially in developing countries. AGPT has significantly enhanced agricultural productivity and reduced environmental pollution^17^, playing an increasingly crucial role in the depth of agricultural industrial chains and supply chains. For instance, converting agricultural food waste into valuable natural pigments not only mitigates environmental burdens but also fosters the development of a green circular economy^18^. By utilising precision irrigation systems, it is possible to ensure that crops receive the right amount of water throughout their growth cycle, thus improving water resource efficiency and crop yields^19^. Sensor technology enables farmers to apply fertilisers and pesticides precisely based on the actual needs of the soil and crops, reducing the excessive use of agrochemicals and lowering environmental pollution^20^. In recent years, the application of information and communication technology (ICT) in agriculture has further advanced the progress of green agricultural development^21^. ICT aids in optimising resource allocation and enhancing production efficiency, contributing to truly sustainable and healthy agricultural practices. Studies indicate that AGPT, through the introduction of innovative technologies and improved resource management efficiency, has significantly increased the sustainability and eco-friendliness of agriculture globally, particularly in developing countries^22^.

Despite comprehensive research and development in AGPT, its application in production practices faces numerous challenges. Firstly, the high costs and technological demands of green agricultural development limit its widespread adoption. A lack of necessary financial and technological support hampers the diffusion of AGPT. Secondly, the implementation of green agricultural technologies requires coordination among various stakeholders, including governments, farmers, businesses, and consumers. However, conflicts of interest and poor communication among these groups often complicate policy implementation^17^. Thirdly, the promotion of AGPT is also constrained by the level of farmers' awareness and acceptance. Many farmers have limited knowledge of new technologies and lack relevant training and education, leading to low acceptance of these technologies^18^. Fourthly, regional differences pose barriers. Variations in climate conditions, soil types, and cultivation structures across regions make it difficult for a uniform AGPT promotion plan to be universally applicable^21^. Fifthly, the development and application of green agricultural technologies often rely on strong capabilities in technological innovation and research and development. However, many countries and regions still fall short in investing in technological innovation and research, limiting the development and dissemination of new technologies. Lastly, the effectiveness of AGPT in practical applications lacks robust evaluation and monitoring mechanisms. The absence of adequate data support and scientific assessment tools makes it difficult to quantify and evaluate the effects of technology applications^22^. Addressing these issues requires multi-faceted collaboration and comprehensive governance, including enhancing technological investment, improving farmers' awareness and acceptance, perfecting policy support and evaluation mechanisms, and considering regional differences, cultural factors, and specific technological influences that affect farmers' actual adoption of technologies.

### Determinants of AGPT adoption willingness

To understand the drivers behind farmers' adoption of AGPT, researchers have collected and analysed a vast array of data related to agricultural production, socio-economic, and environmental factors. Scholars have found that the reasons influencing farmers' adoption of AGPT are affected by a combination of internal and external factors. Internal factors such as education level, farmland area, and income level^23–25^ play a crucial role in shaping farmers' perceptions and attitudes towards new technologies. These differences in cognition ultimately impact their willingness to adopt new agricultural technologies. Kassem et al.^26^ divide the technology adoption process into five stages: awareness, interest, evaluation, trial, and adoption. Based on these stages, the impact of internal factors on AGPT adoption behaviour can be summarized into three categories: information acquisition capability, technology cognition ability, and risk-taking ability^27^. In terms of information acquisition capability, among farmers with several family members working away from home, obtaining information on new technologies from these members helps reduce resistance within the family and enhances recognition of the economic and environmental benefits of AGPT, thereby increasing the willingness to adopt new technologies^28^. Farmers with larger production scales have a greater capability to understand technology, placing more emphasis on the convenience brought by new technologies and the reduction in labour costs, hence showing a higher acceptance of AGPT^29^. Regarding technology cognition ability, research by McCarthy and Liu indicates that as government publicity and public environmental awareness increase, more consumers opt for green organic products when making purchases^30,31^. If farmers believe that AGPT can improve both production efficiency and the quality of agricultural products, as well as effectively utilize resources and protect the environment, they are more likely to adopt this new technology^32^. Study on the impact of risk-taking ability has found that farmers’ awareness of personal risks (health, environment, and product safety) also plays an active role in promoting the implementation of low-carbon behaviours^33^. For example, a study on farmers in Punjab, Pakistan, finds that changes in agricultural policy were identified as the greatest source of risk^34^. Another study in the northeastern United States indicates that business pressures, such as profitability, market conditions, labour supply, or government regulations, are often the most critical issues affecting farmers' decisions^35^.

External factors can be categorized into three groups based on their functional mechanisms and areas of impact: information sources, government regulation, and market conditions^36–38^. Firstly, the quality and availability of information determine farmers' level of understanding and willingness to accept new technologies^39^. Studies have shown that farmers' technology adoption behaviours exhibit a notable clustering tendency^40^. After farmers join cooperatives, the positive information gathered during social interactions helps reduce their perceived risk of AGPT and increases their willingness to adopt it^41^. High-quality information sources help farmers better understand and apply AGPT, enhancing agricultural production efficiency^42^. Additionally, research findings indicate that both media channels and social interaction significantly impact farmers adoption of straw return techniques, with these two channels having a mutually reinforcing effect^43^. Secondly, governments influence farmers' attitudes and behaviours towards AGPT directly or indirectly through policy formulation, financial support, and educational campaigns. For instance, internet-based environmental protection and green agriculture policy publicity have a significant direct effect on reducing farmers' use of pesticides^44^. Promoting the environmental benefits and economic incentive policies of renewable energy through social media can positively regulate farmers' willingness to adopt biogas technology^45^. Lastly, economic factors such as demand for agricultural products, trade patterns, and pricing mechanisms indirectly encourage farmers to adopt environmentally friendly production technologies by influencing their economic benefits and market competitiveness^35^. For example, as farmers trade agricultural products online through platforms owned by Alibaba, the increasing awareness of building a quality traceability system and honesty in transactions has led to a market-driven reduction in pesticide use^46^.

In summary, extant research has thoroughly investigated the influence of technological, economic, and environmental factors on AGPT adoption from both internal and external perspectives. Despite this extensive exploration, notable gaps persist within the existing literature. Firstly, there is a relative scarcity of studies on how regional culture and social structures influence AGPT adoption. This lack of region-specific analysis may lead to an incomplete understanding of the motivations and barriers faced by farmers in different areas when adopting new technologies. Secondly, while previous research has identified the importance of information sources and policy support, there is insufficient investigation into how these factors specifically affect different types of agricultural households. Lastly, there is a lack of in-depth empirical research exploring the relationship between technological innovation and the adoption of AGPT. To address these deficiencies, this study introduces an extended technology acceptance model to analyse how internal and external factors jointly influence the adoption of AGPT. Moreover, a case study was conducted in a representative province of China to examine how local culture and the quality of different information sources affect farmers' acceptance and adoption of AGPT.

## Methodology and data

### TAM

**Figure 1:**
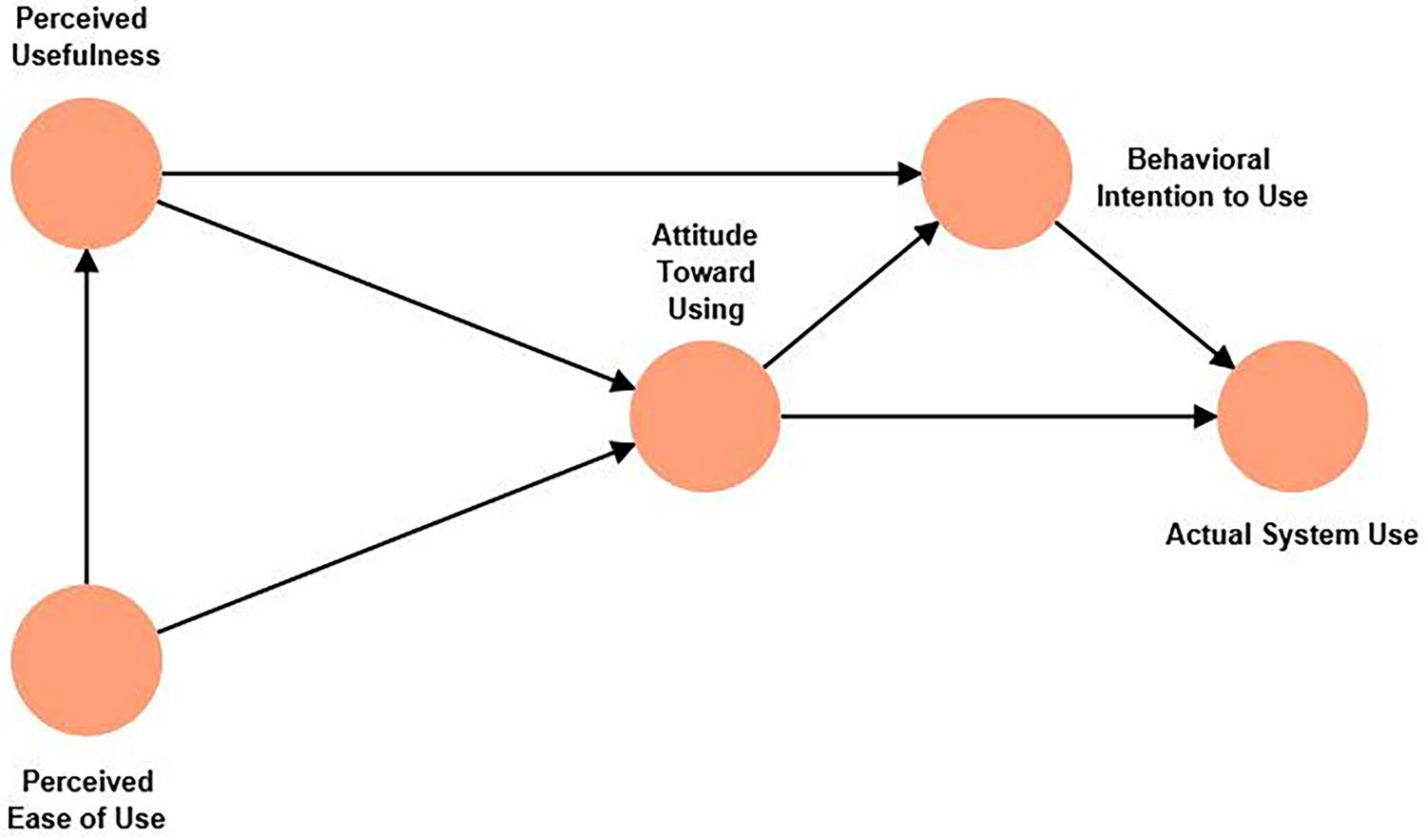
TAM framework. This figure represents the structural diagram of the technology acceptance model (TAM), which elucidates the causal relationships between system design features, perceived usefulness, perceived ease of use, attitude toward using, behavioral intention to use, and actual system usage.

Based on the theory of reasoned action and social and cognitive psychology, Davis^47^ proposes the technology acceptance model (TAM) to predict people's acceptance and intention to use new technologies. The TAM identifies two primary determinants: perceived ease of use (PEOU) and perceived usefulness (PU). PEOU refers to an individual's feelings about using new technology, including the difficulty of learning and the ease of operation of the technology. If a new technology is easy to use, individuals are more likely to accept and use it. PU pertains to an individual's cognitive assessment of whether new technology can bring real value and benefits. If individuals believe that new technology is helpful for them, they are more likely to accept and use it. PEOU and PU are influenced by a variety of factors at both the individual and social levels, including basic user characteristics (age, education level, etc.), policy norms, environmental constraints, and more^48–50^. These factors collectively affect individuals' perceptions of AGPT, thereby influencing their acceptance of and intention to use new technologies^51^ (as shown in Fig. 1).

In other commonly used theoretical methods for studying factors influencing technology adoption, innovation diffusion theory (IDT) focuses on the process of the spread of new technologies or innovations over time within a specific social system, emphasizing the interaction between technological characteristics and the sociocultural environment^52^. The theory of planned behaviour (TPB) concentrates on predicting and understanding individual behaviour by considering the attitudes, subjective norms, and perceived behavioural control of individuals^53^. Compared to these models, the TAM's advantage lies in its focus on the characteristics of the technology itself (ease of use and usefulness) and the direct impact of these characteristics on users' willingness to accept, making it more concise and effective for specific technology adoption studies.

Due to the simplicity, reliability, operability, and interdisciplinary applicability of the TAM, it has been widely applied across various industries to predict different groups' acceptance of various new technologies. For example, in the health care sector, the TAM has been used to predict patients' acceptance of telemedicine services^54^. In the field of education, the TAM has been used to predict the extent to which teachers and students accept online teaching tools^55^. In the business sector, it has been employed to predict consumers' acceptance of live shopping^56^. In agriculture, the TAM can also be used to predict farmers' willingness to accept technology. Dai et al.^30^ add perceived value to the TAM to analyse the driving factors for farmers' adoption of green production technologies. Based on the TAM, Aubert et al.^57^ analyse the promotional effects of information technology on farmers' decisions to adopt precision agriculture technologies. Savari et al.^58^ use the TAM to study the determinants of environmentally friendly behaviours among Iranian farmers. Dong et al.^59^ combine the TAM and the theory of planned behaviour to predict factors affecting Chinese farmers' willingness to adopt rice-shrimp cropping technology. Following these applications of the model, this study adopts the TAM to predict and analyse the factors influencing farmers' intentions to adopt AGPT in Liaoning Province, China.

### Model development

#### Perceived ease of use

According to Davis's definition of the TAM, in predicting the intention to adopt AGPT, perceived ease of use (PEOU) refers to whether farmers find the technology simple, convenient, and easy to operate. Research from Italian scholars^42^ indicates that PEOU significantly impacts farmers' intention to adopt sustainable production technologies. Compared to more familiar traditional production technologies, if the operation of AGPT is overly complicated, farmers may lose confidence in learning the technology, and vice versa. Therefore, we propose the following hypothesis:

##### Hypothesis 1

PEOU positively influences farmers' intention to adopt AGPT.

#### Perceived usefulness

According to the model's definition, in analysing farmers' intentions to adopt AGPT, perceived usefulness (PU) refers to the efficiency gains of green production technology over traditional production methods, such as reducing resource waste and increasing yield or bringing additional benefits, such as enhancing the quality and sales of agricultural products. The use of scientific irrigation and reasonable crop rotation, among other low-carbon production technologies, can accelerate crop growth and increase yields^60^, while the prudent use of organic pesticides and fertilizers aids in the production of healthier and safer agricultural products^61^. Therefore, we propose the following hypothesis:

##### Hypothesis 2

PU positively influences farmers' intention to adopt AGPT.

#### Perceived risks

Although the TAM has been validated in various fields for predicting the acceptance of new technologies, incorporating new influencing factors into the original model can further enhance its predictive accuracy. When introducing new technologies, an individual's decision to adopt the technology is influenced by various objective risk assessments and personal attitudes, which in turn affect their decision-making. For example, Pavlou et al.^62^ find that perceived risk is one of the key drivers affecting consumers' participation in online transactions. In terms of agricultural production, research by Asravor et al.^63^ confirms that the perception of market risk dramatically influences the use of improved seed varieties and the application of inorganic fertilizers. Therefore, we incorporate perceived risk (PR) into the TAM, combined with PEOU and PU, to help predict farmers' willingness to adopt AGPT.

According to the extended technology acceptance model, PR refers to the perception of risks related to climate change, soil quality, market demand, and the psychological ability to assess the impact of using AGPT on the environment and economy. When adopting new production technologies, farmers must consider the additional time required to learn the technology and the demand for procurement funds, which, under the dual pressures of time and economic costs, can also face opposition from family members^64^. Consequently, the greater the intensity of PR is, the lower the enthusiasm farmers have towards adopting AGPT. Based on this, we propose the following hypothesis:

##### Hypothesis 3

PR negatively influences farmers' intention to adopt AGPT.

#### Information acquisition

In the era of emerging information technologies, digital multimedia technologies carried by the internet have substantially broadened the channels through which farmers acquire information, playing an essential role in breaking down knowledge barriers and reducing information costs^65^. Digital multimedia systems transmit and enhance information through various formats, such as images, audio, and video, offering robust interactivity in knowledge dissemination. Smith et al.^66^ conclude from their analysis of survey data on farmers in the Great Plains of the United States that farmers' production decisions and income are closely related to the extent of internet applications. In the learning process of AGPT, farmers can not only learn about related agricultural information technologies but also become aware of green production and improve their literacy in modern agricultural production^67^. They can continue to adopt green technologies, forming a closed loop of green production. The more content farmers acquire related to green production through digital multimedia, the more likely they are to adopt similar technologies in subsequent production. Hence, we propose the following hypothesis:

##### Hypothesis 4

Information acquisition through digital multimedia positively influences farmers' intention to adopt AGPT.

The universality, permeability, and interactivity of digital multimedia facilitate the dissemination of information and play a vital role in spreading rural ecological innovations and influencing farmers' pro-environmental behaviours. Disseminated through digital multimedia, videos about environmental pollution can help farmers recognize agricultural pollution issues and the negative impacts of traditional agricultural technologies, thereby enhancing their awareness of the environmental benefits of AGPT. Moreover, watching learning videos related to AGPT can help reduce the psychological difficulty of using new technologies. For instance, a randomized controlled field experiment in Burkina Faso^68^ assesses the effectiveness of animated videos played on mobile phones compared to traditional extension methods (live demonstrations) in inducing low-literacy farmers to learn and adopt pesticide reduction and pest control techniques. Additionally, digital platforms such as social media provide free space for farmers to interact with and discuss AGPT-related issues, which can better assist in mastering new technologies and thus reduce perceived risks. Therefore, we propose the following hypotheses:

##### Hypothesis 4a

Information acquisition through digital multimedia positively influences farmers' intention to adopt AGPT by affecting PEOU.

##### Hypothesis 4b

Information acquisition through digital multimedia positively influences farmers' intention to adopt AGPT by affecting PU.

##### Hypothesis 4c

Information acquisition through digital multimedia positively influences farmers' intention to adopt AGPT by affecting PR.

#### Digital marketing

With increasing global attention given to climate change, the digital economy has become a new pathway for driving the decarbonization of industries. In agriculture, promoting the integration of digitalization and the agricultural economy can further enhance production efficiency^69^ and accelerate green development in the industry. In the process of digitizing the agricultural marketing system, in addition to using digital multimedia to acquire information resources, an increasing number of farmers are choosing to use internet platforms for the promotion and advertisement of agricultural products. Digital marketing^70^ is a form of marketing that utilizes the internet and digital technologies to promote products and services, establishing connections with current and potential customers through various online channels to enhance brand awareness, increase sales, and improve customer experience. Online marketing of agricultural products can eliminate the drawbacks of traditional marketing methods, bypass intermediaries, and enable direct contact between farmers and consumers, thus minimizing the stages and costs involved in the entire transaction process^71^. Research^72^ on Chinese agricultural e-commerce, particularly through the use of digital multimedia technologies such as short video live streaming, has revealed a transformative impact on the marketing and consumption of agricultural products. This mode of presentation significantly enhances consumer awareness of and interest in products with pro-environmental features, such as green cultivation practices. By effectively harnessing the interactivity and real-time capabilities of digital multimedia, agricultural practitioners can promote sustainable agricultural practices more broadly. Therefore, we propose the following hypothesis:

##### Hypothesis 5

Digital marketing positively influences farmers' intention to adopt AGPT.

With the deepening use of digital multimedia, farmers' online sales capabilities are continuously strengthened, and awareness of food safety and efficiency in the circulation of agricultural products is enhanced, gradually leading to green production consciousness. This change leads to the realization of the importance of adopting AGPT^73^. Furthermore, digital marketing platforms offer high levels of transparency, calling for farmers to share comprehensive details about environmentally friendly agricultural products^74^. This information includes the underlying technological principles and practical use cases. Such transparency can help regulate the implementation of AGPT by farmers and alleviate any apprehensions that potential adopters may have about the safety and dependability of the technology. Therefore, we propose the following hypotheses:

##### Hypothesis 5a

Digital marketing positively influences farmers' intention to adopt AGPT by affecting PEOU.

##### Hypothesis 5b

Digital marketing positively influences farmers' intention to adopt AGPT by affecting PU.

##### Hypothesis 5c

Digital marketing positively influences farmers' intention to adopt AGPT by affecting PR.

**Figure 2:**
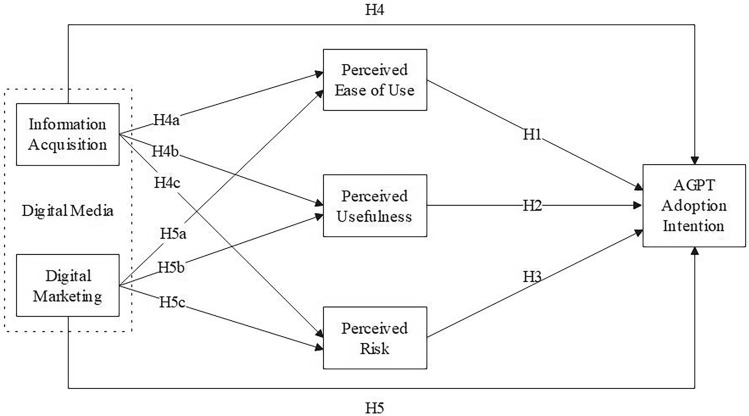
Research model. This figure illustrates a research framework aimed at understanding the factors that influence farmers' decision to adopt agricultural green production technology (AGPT). The model identifies perceived ease of use (PEOU), perceived usefulness (PU) and perceived risk(PR) as key factors that impact farmers’ intentions to adopt AGPT. Information acquisition (IA) from digital multimedia sources and digital marketing (DM) initiatives are seen as influential in shaping PEOU, PU, and PR, which in turn affect the adoption intention. The hypothesis H4 posits that IA has an influence on adoption intention, while H4a, H4b, and H4c suggest that this effect is mediated through its impact on PEOU, PU, and PR, respectively. Similarly, H5 supports the direct influence of DM on adoption intention, with H5a, H5b, and H5c indicating mediation through PEOU, PU, and PR. The structure of this model illustrates the flow of influence from the information and marketing strategies employed, through the perceptions of the technology, to the ultimate adoption decision by farmers. The arrows denote the hypothesized pathways of influence.

In summary, this study builds an extended technology acceptance model by adding the variable of perceived risk to the TAM and considering digital multimedia to explore the factors affecting farmers' adoption of AGPT. Based on the above hypotheses, we propose a new research model to explain the intention to adopt AGPT (as shown in Fig. 2).

### Data sources

#### Data collection

The research design received approval from the Ethics Committee of the School of Information and Business Management at Dalian Neusoft University of Information. In strict compliance with the Helsinki Declaration, our study was conducted following all pertinent guidelines and regulations. Informed consent was obtained from each participant, who were all duly informed about the purpose of the study and assured of the survey's anonymity. The outcomes of this research will be exclusively utilized for scientific purposes, with a firm commitment to maintaining the confidentiality of personal information.

Liaoning Province, located in northeastern China, features geographic characteristics related to the transition from land to sea. With fertile soil and a climate conducive to a variety of crops, Liaoning Province is one of China's major agricultural regions. The province primarily focuses on grain production, with a sowing area of 53.423 million mu. In 2021, the total grain output reached 23.024 billion kilogram, making Liaoning a leading grain-producing province in China. The government of Liaoning Province has been taking steps to encourage the implementation of environmentally friendly, high-quality, and efficient techniques for grain production in recent years. This initiative is mainly focused on rice and corn, with the aim of promoting conservation tillage techniques and achieving large-scale, balanced yield increases. Therefore, selecting rice and corn growers from Liaoning Province as a survey sample is highly representative.

The data uses in this study were obtained from field surveys conducted by the research team from April to June 2023. Based on the geographical division characteristics of Liaoning Province, four major grain-producing regions are chosen for the study: northern Liaoning, central Liaoning, western Liaoning, and southern Liaoning. A sampling method combining stratified sampling with simple random sampling is used. Two sample counties are selected from each region, and 2–4 townships are chosen from each sample county. Two to three villages are selected from each township. In the actual survey, farmers are randomly selected from each sample village for face-to-face interviews. The survey is conducted using questionnaires and included two parts: basic statistical characteristics of the farmers and factors influencing their intention to adopt green production technologies. To ensure consistent data collection, the research team trains surveyors uniformly beforehand. Participants are informed that the questionnaire is anonymous and confidential, and incomplete or logically flawed questionnaires are excluded. A total of 500 questionnaires are distributed in this study, with 458 valid responses, for an effective response rate of 91.6%.

#### Data analysis

The questionnaire-referenced measurement items have been validated in previous studies and were adjusted according to China's specific agricultural environment and grain planting background (as shown in Table 1).

**Table 1:** Measurement items.

Variable	Indicator	Item	Origin
Perceived usefulness	PU1	I believe using AGPT can reduce production costs	Xiang et al.^75^
	PU2	I believe using AGPT can protect the environment and reduce pollution	
	PU3	I believe using AGPT can increase financial subsidies for products	
Perceived ease of use	PEOU1	I find AGPT easy to implement	Dai et al.^37^
	PEOU2	I find it easy to access information about AGPT	
	PEOU3	I think learning AGPT is not difficult	
Perceived risk	PR1	I believe using AGPT requires more financial investment	Li et al.^11^
	PR2	I believe learning AGPT requires a lot of time	
	PR3	I believe using AGPT may not be accepted by family or community	
Information acquisition	IA1	I learned about government support for AGPT through digital multimedia	Zhao et al.^44^
	IA2	I saw information about AGPT training on digital multimedia platforms	
	IA3	I saw information about environmental protection on digital multimedia platforms	
Digital marketing	DM1	I learned about the high consumer demand for green agricultural products through e-commerce platforms	Tie et al.^76^
	DM2	I learned about strict quality standards for green agricultural products on e-commerce platforms	
	DM3	I believe promoting agricultural products on digital multimedia platforms can increase sales	
AGPT adoption intention	AI1	My intention to adopt diversified crop rotation	Ren et al.^38^
	AI2	My intention to adopt organic farming	
	AI3	My intention to adopt straw return to the field	

An expert group consisting of two professors, two agricultural managers, and two agricultural practitioners is invited to discuss each measurement item individually, ensuring the content validity of the questionnaire and improving the precision of the scale. Considering that the respondents are generally older, a 7-point Likert scale might cause ambiguity. Therefore, participants are instructed to use a 5-point Likert scale to rate each item on a scale ranging from 1 (strongly disagree) to 5 (strongly agree).

In this study, the statistical analysis software SPSS and the partial least squares structural equation modelling software SmartPLS 4.0 are utilized to analyse the questionnaire data. Structural equation modelling (SEM) is employed to examine the path relationships between variables and the effect of latent variables in the model. Compared to other methods, SEM provides a more comprehensive framework for hypothesis testing and model assessment. It not only predicts direct and indirect impacts but also analyses latent relationships between variables, presenting them intuitively through path diagrams^77^.

Initially, frequency and percentage are used to describe the characteristic statistical data, followed by testing the reliability, convergent validity, and discriminant validity of the scale. Reliability testing reflects the internal consistency of the measurement items. We use Cronbach's α and composite reliability (CR) for the assessment. Higher values indicate better internal consistency of the scale, with values greater than 0.7 considered acceptable. Convergent validity refers to the degree of association between two or more variables. It is tested using factor loadings and average variance extraction (AVE) of the measurement items, with values greater than 0.5 passing the convergent validity test^78^. Discriminant validity tests the degree to which different constructs in the scale can be clearly distinguished. If the square root of a construct's AVE is greater than its correlation with any other construct, it is considered to have good discriminant validity^78^. Finally, the model's fit is tested. When the ratio of chi-square to degrees of freedom (X2/DF) is less than 5, the root mean square error of approximation (RMSEA) is less than 0.1, and the values of the comparative fit index (CFI), incremental fit index (IFI), goodness-of-fit index (GFI), normed fit index (NFI), Tucker‒Lewis index (TLI), and adjusted goodness-of-fit index (AGFI) are close to 1, indicating a better model fit^79^. Following the assessment of the model's fit, path coefficients are examined for statistical significance at thresholds of p < 0.001, p < 0.01, and p < 0.05, with lower p-values indicating stronger evidence against the null hypothesis. T-values are used to determine the reliability of these coefficients, where higher values indicate greater statistical significance of the relationships in the model. A t-value of about 2 generally signifies significance at the p < 0.05 level, while higher thresholds require correspondingly larger t-values^79^.

## Results

### Descriptive statistics

As shown in Table 2, 458 rural households completed the questionnaire survey. The sample characteristics are broadly consistent with the regional field research situations, demonstrating good representativeness. In the sample, male farmers constitute 67.69%, while female farmers are in the minority. The age structure of the sample is mainly composed of middle-aged and older individuals. The most represented age group is 50 to 60 years, accounting for 34.72%, followed by the 40 to 50 year age group, which accounts for 22.71%. In terms of educational level, 33.84% of the farmers have a high school education, followed by 30.13% who have a primary school education or below. There are relatively few farmers with a college education or higher, making up only 7.42% of the total sample. The distribution of the family labour force shows that most rural households have a labour force of 3 to 5 people, accounting for 35.15% of households surveyed. In terms of cultivable land area, households with more than 10 acres of land constitute the largest proportion, at 33.41%. The annual household income data indicates that 30.35% of the households have an income between 70,000 and 100,000 yuan, while those earning more than 100,000 yuan account for only 16.16%.

**Table 2:** Demographic characteristics of participants.

Characteristics	Category	Frequency
Gender	Female	148
	Male	310
Age	(18, 30]	46
	(30, 40]	67
	(40, 50]	104
	(50, 60]	159
	> 60	82
Education level	Elementary school or below	138
	Middle school	131
	High school	155
	Associate degree	27
	Bachelor degree or above	7
Household labor force	(1, 3]	58
	(3, 5]	161
	(5, 7]	145
	> 7	94
Arable area (mu)	(1, 3]	82
	(3, 5]	119
	(5, 10]	104
	> 10	153
Annual household income (10, 000 yuan)	(3, 5]	120
	(5, 7]	125
	(7, 10]	139
	> 10	74

### Reliability and validity test

The results of the questionnaire validation indicate that the scales used perform well in terms of reliability and validity (as shown in Table 3). Specifically, the Cronbach α values for each construct range from 0.76 to 0.85, demonstrating high internal consistency. The composite reliability (CR) also shows strong reliability, with values ranging from 0.71 to 0.88, further confirming the reliability of the scales. The average variance extracted (AVE) values range from 0.51 to 0.68, with most constructs exceeding the recommended threshold of 0.5, indicating good convergent validity of the questionnaire. The factor loadings of each measurement item are between 0.61 and 0.88, suggesting that each measurement item has high explanatory power for its respective construct. Notably, for the perceived risk (PR) and information acquisition (IA) constructs, the third measurement items have factor loadings of 0.81 and 0.84, respectively, the highest among the constructs, showing strong indicator relevance. In summary, the scale demonstrates good reliability and validity in measuring the intended multiple latent variables, providing solid support for subsequent model validation.

**Table 3:** Reliability and validity test results.

Constructs	Items	Factor loading	Cronbach's α	CR	AVE
PEOU	PEOU1	0.80	0.81	0.71	0.51
	PEOU2	0.78			
	PEOU3	0.65			
PU	PU1	0.62	0.76	0.88	0.65
	PU2	0.77			
	PU3	0.64			
PR	PR1	0.64	0.79	0.84	0.68
	PR2	0.76			
	PR3	0.81			
IA	IA1	0.64	0.85	0.85	0.61
	IA2	0.62			
	IA3	0.84			
DM	DM1	0.61	0.76	0.76	0.59
	DM2	0.77			
	DM3	0.88			
AGPT AI	AI1	0.79	0.72	0.80	0.68
	AI2	0.61			
	AI3	0.70			

### Hypothesis test

The model's fit was assessed using SmartPLS 4.0, and the results are presented in Table 4. The model achieves or exceeds the acceptance criteria across various key indicators, indicating a high degree of consistency between the model's structure and the observed data. Therefore, from a statistical perspective, this model is effective and reliable, providing a solid foundation for subsequent analyses.

**Table 4:** Model fit assessment results.

Model indicators	X2/DF	RMSEA	CFI	IFI	GFI	NFI	TLI	AGFI
Criteria	< 5	< 0.10	> 0.90	> 0.90	> 0.90	> 0.90	> 0.90	> 0.90
Fit index	3.967	0.085	0.908	0.927	0.939	0.927	0.945	0.923

The results of the hypothesis testing and the path coefficients (β) are presented in Table 5. The influences of perceived ease of use (PEOU) and perceived usefulness (PU) on the intention to adopt agricultural green production technology (AGPT) are significant (β = 0.374, p < 0.001; β = 0.302, p < 0.01), indicating that farmers' technological cognition of AGPT plays a crucial role in their decision-making process. Concurrently, perceived risk (PR) shows a significant negative correlation with the intention to adopt AGPT (β = − 0.244, p < 0.01), suggesting that farmers' perceptions of the risks associated with adopting AGPT hinders their willingness to adopt it. Hence, Hypotheses H1, H2, and H3 are supported. Based on the rational economic person assumption, farmers prioritize benefits in decision-making^63^. Due to insufficient technological cognition, risk-averse farmers tend to stick to familiar traditional production methods when facing new technologies^73^. In contrast, farmers with better risk acceptance under external influences, when evaluating new technologies, are more willing to try and adopt these technologies if they believe they will provide higher average profits than existing methods.

**Table 5:** Hypothesis test results.

Assumed path	Standardization coefficient	S.E.	t value
PEOU → AI	0.374**	0.043	8.698
PU → AI	0.302*	0.051	5.922
PR → AI	− 0.244*	0.047	− 5.191
IA → AI	0.268*	0.056	4.786
IA → PEOU	0.128*	0.032	4.000
IA → PU	0.225*	0.042	5.357
IA → PR	0.009	0.041	0.220
DM → AI	0.228*	0.061	3.738
DM → PEOU	0.474**	0.041	11.561
DM → PU	0.208*	0.056	3.714
DM → PR	− 0.343*	0.066	− 5.197

**Indicates p < 0.001, *Indicates p < 0.01.

Further analysis reveals that information acquisition through digital multimedia has a significant positive impact on the intention to adopt AGPT (β = 0.268, p < 0.01). Additionally, digital multimedia information acquisition positively influences both PEOU (β = 0.128, p < 0.01) and PU (β = 0.225, p < 0.01), aligning with the expectations of Hypotheses H4a and H4b. However, the impact of information acquisition (IA) on PR is not significant (β = 0.009); thus, Hypothesis H4c is not supported. The habit of frequently using digital multimedia can increase farmers' sensitivity to external information, enabling them to stay informed about the latest policies and technological trends and continually enhancing their technology cognition and green production consciousness. However, older farmers or those with lower educational levels tend to exhibit reduced trust in information disseminated through online channels^45^. This skepticism hampers their ability to alleviate concerns about adopting new production technologies based solely on the information acquired online.

Furthermore, the study reveals a positive impact of digital marketing on the intention to adopt AGPT (β = 0.228, p < 0.01). Digital marketing significantly enhances PEOU (β = 0.474, p < 0.001) and PU (β = 0.208, p < 0.01) and significantly reduces PR (β = − 0.343, p < 0.01). These results suggest that Hypotheses H5, H5a, H5b, and H5c are supported. Products produced using green production technologies can usually be sold as high-quality goods, thus obtaining higher added value^80^. Digital marketing methods can increase farmers’ awareness of the economic benefits of AGPT, increasing their willingness to adopt it. Additionally, social media and other digital marketing channels can facilitate interaction and communication among farmers, enhancing a sense of identity and mimetic effects within the group^81^ and thereby increasing their willingness to adopt AGPT.

We employ the bias-corrected bootstrap method to estimate the 95% confidence intervals (CIs) and analyse the indirect effects of information acquisition (IA) and digital marketing (DM) on the intention to adopt AGPT. We use IA and DM as independent variables; perceived ease of use (PEOU), perceived usefulness (PU), and perceived risk (PR) as mediating variables; and the intention to adopt AGPT as the dependent variable, and estimations are based on 5000 bootstrap samples. According to the results of the indirect effect test (shown in Table 6), the indirect effect of information acquisition through PU on the intention to adopt AGPT is 0.0475, and that through PEOU is 0.0682. These results indicate that PU and PEOU play significant mediating roles in the impact of information acquisition on the intention to adopt AGPT, thus supporting Hypotheses H4a and H4b. However, the indirect effect of information acquisition through PR on the intention to adopt AGPT is − 0.0022, with a confidence interval of [− 0.0221, 0.0184]. This result suggests that perceived risk did not significantly mediate the impact of IA on the intention to adopt AGPT, and Hypothesis H4c is not supported. Regarding digital marketing, the indirect effect of PEOU on the intention to adopt AGPT is 0.1773, that through PU is 0.0627, and that through perceived risk is 0.0841. The test results confirm that PEOU, PU, and PR all play mediating roles in the impact of digital marketing on the intention to adopt AGPT, further supporting Hypotheses H5a, H5b, and H5c.

**Table 6:** Indirect effect test results.

Independent variable	Mediator	Indirect effect	LLCL	ULCL
IA	PEOU	0.0475	0.0237	0.0744
IA	PU	0.0682	0.0381	0.1055
IA	PR	− 0.0022	− 0.0221	0.0184
DM	PEOU	0.1773	0.1279	0.2304
DM	PU	0.0627	0.0268	0.1050
DM	PR	0.0841	0.0446	0.1342

In summary, according to the results of the path analysis, Hypotheses H1, H2, H3, H4, and H5 are supported. Based on the results of the mediation effect analysis, Hypotheses H4a, H4b, H5a, H5b, and H5c are supported, whereas Hypothesis H4c is not supported.

## Discussion and implications

### Discussion

This study examines the decision-making factors influencing grain farmers' adoption of AGPT based on the extended TAM. We propose research hypotheses and analyse the relationship between the use of digital multimedia and farmers' intention to adopt AGPT. Through the analysis of field survey data from rice and corn farmers in Liaoning Province, it is confirmed that perceived ease of use (PEOU), perceived usefulness (PU), and perceived risk (PR) directly impact farmers' intention to adopt AGPT, which is consistent with previous studies on farmers' adoption of new production technologies^11,37^.

Furthermore, our research findings indicate that information acquisition through digital multimedia is positively correlated with PEOU and PU, thereby indirectly influencing their willingness to adopt AGPT. This finding aligns with previous research suggesting that internet usage promotes farmers' choices to reduce pesticide use^44^. Additionally, our study explores the impact of PR in the information transmission process of digital multimedia on farmers' adoption of AGPT and its role as a mediating factor. However, contrary to conclusions in the previous literature^65^, our study results indicate that this mediating effect is not significant. The main reason is that digital multimedia is an emerging information dissemination platform, and older farmers are more inclined to rely on their social networks, taking advice from village leaders or family members when making technology adoption decisions^45^. This behaviour suggests that in promoting green agricultural production technologies, in addition to utilizing digital multimedia, the educational background and social trust structure of rural communities should also be considered.

Finally, our study reveals that digital marketing has a significant positive impact on the willingness to adopt AGPT through PEOU, PU, and PR. Promoting and selling agricultural products through online platforms encourages farmers to choose green agricultural technologies for production. This aligns with the view from previous research that digital marketing platforms can also serve as platforms for agricultural technology education and training^43^. Through video tutorials, online seminars, and other digital multimedia materials, farmers can more directly understand the application and benefits of AGPT. Moreover, various incentive policies on these platforms can provide farmers with a more direct understanding of the government's financial support for the production and sale of green agricultural products. Collectively, these factors encourage farmers to actively adopt AGPT to better meet market demand and policy directives.

### Practical implications

Based on the empirical research data and analysis of the results, we propose the following interventions to enhance farmers' intention to adopt green production technologies: First, the government should utilize digital multimedia platforms to invite agricultural experts or successful practitioners of AGPT for technical explanations and experience sharing. Compared to traditional information channels, digital multimedia can present technology more vividly and intuitively, making it easier for farmers with lower education levels to understand and accept it, thereby translating into behavioural responses^82^. Previous studies have confirmed the effectiveness of digital platforms in disseminating information and exerting influence^65^. However, our results indicate that this approach is not sufficient to alleviate the concerns of technology adopters about potential risks directly. Therefore, the government can organize online Q&A sessions, interactive live broadcasts, and virtual seminars on digital multimedia platforms such as TikTok, allowing farmers to interact directly with AGPT experts and successful practitioners, posing their questions and concerns. This interactive approach not only helps provide authoritative and practical technical information but also enhances farmers' trust in and acceptance of AGPT.

Second, government departments can offer professional training in digital marketing methods to farmers intending to adopt AGPT technology. Our study results indicate that practical experience in digital marketing has a positive impact on both enhancing AGPT technology cognition and reducing risks. Increasing support for agricultural e-commerce can not only enhance the market value of green agricultural products but also promote green production technologies. For instance, providing farmers with professional training for live broadcasting, guiding them to highlight the green production characteristics of their products during marketing, and determining how to interact with potential consumers can increase their sales potential. Additionally, encouraging the creation of new media account matrices at the village or township level can enhance cooperation between villages and towns, promote the sharing of AGPT-related knowledge and resources, and showcase AGPT practices and outcomes to a broader audience.

Third, agricultural managers should conduct targeted AGPT training by organizing community meetings. Specifically, concentrated training for village officials or family representatives can be provided to disseminate critical information, and early adopters can be cultivated to promote the use of AGPT. According to our study results, obtaining technical information from the internet does not significantly impact the reduction of risk perception. However, this cascading method can not only enhance farmers' perception of the ease of use of AGPT but also leverage established social trust networks to increase technology acceptance and reduce risk perception. Moreover, this study recommends that AGPT technology developers optimize and improve existing technologies, including simplifying the technological operation process and reducing complexity, as well as providing more intuitive and easy-to-understand operation guides to cater to the needs of farmers with different educational backgrounds. The study results show that PEOU, PU, and PR significantly impact the intention to adopt technology, and simplifying the AGPT process helps not only in reducing psychological and operational barriers for farmers in adopting new technology but also in increasing the popularity and effectiveness of technology.

## Conclusions

This study applies the TAM to the field of agriculture and introduces a new variable, perceived risk, to the original model, constructing an extended technology acceptance model. This modification provides a new perspective for theoretical research in the field of agricultural technology acceptance. The results show that digital marketing positively influences the willingness to adopt AGPT through PEOU, PU, and PR. However, in the mediation effect test of digital multimedia information acquisition on technology adoption intention, the mediating role of PR is not significant. This finding indicates that while digital multimedia can effectively enhance the dissemination and understanding of technology, its role in reducing farmers' concerns about adopting AGPT technology is limited. In the empirical survey, unlike most studies focusing on specific technologies, our research, based on existing measurement items, comprehensively examines green production technologies in all stages of grain production (pre, during, and post). The results are more general and applicable, providing a more comprehensive theoretical basis for the promotion and implementation of AGPT. These findings have meaningful practical implications for guiding policy-makers and practitioners in using digital multimedia to promote sustainable agricultural technology innovation.

This study extensively explores the impact of digital multimedia on information acquisition and marketing in influencing farmers' adoption of AGPT, addressing a gap in existing research on green production technology adoption. However, it is important to acknowledge certain inherent limitations within the scope of the study. Firstly, the sample data are derived from a single province, and the results are strongly linked to the regional and cultural context of the area. Secondly, the survey participants are limited to farmers growing corn and rice, without considering the potential variability in outcomes due to differences in crops. Thirdly, the study utilizes cross-sectional data, capturing a static moment in history, which does not effectively reveal the trends over time in farmers' willingness to adopt AGPT. Future research aims to expand the sampling scope and scientifically assess the applicability of the findings on a broader scale. Additionally, employing dynamic panel data will ensure a closer alignment of the research outcomes with real-world issues, providing a more comprehensive and profound understanding for the promotion and implementation of AGPT.

## Data Availability

The datasets used and analysed during the current study are available from the corresponding author on reasonable request.
